# Exclusion-Proneness in Borderline Personality Disorder Inpatients Impairs Alliance in Mentalization-Based Group Therapy

**DOI:** 10.3389/fpsyg.2018.00824

**Published:** 2018-05-28

**Authors:** Sebastian Euler, Johannes Wrege, Mareike Busmann, Hannah J. Lindenmeyer, Daniel Sollberger, Undine E. Lang, Jens Gaab, Marc Walter

**Affiliations:** ^1^Department of Psychiatry (UPK), University of Basel, Basel, Switzerland; ^2^Research Department of Clinical, Educational and Health Psychology, University College London, London, United Kingdom; ^3^Department of Counseling and Clinical Psychology, Teachers College, Columbia University, New York, NY, United States; ^4^Psychiatric Baselland, Liestal, Switzerland; ^5^Division of Clinical Psychology and Psychotherapy, Faculty of Psychology, University of Basel, Basel, Switzerland

**Keywords:** borderline personality disorder, Cyberball, social exclusion, Need Threat Scale, mentalization-based group therapy, therapeutic alliance

## Abstract

Interpersonal sensitivity, particularly threat of potential exclusion, is a critical condition in borderline personality disorder (BPD) which impairs patients’ social adjustment. Current evidence-based treatments include group components, such as mentalization-based group therapy (MBT-G), in order to improve interpersonal functioning. These treatments additionally focus on the therapeutic alliance since it was discovered to be a robust predictor of treatment outcome. However, alliance is a multidimensional factor of group therapy, which includes the fellow patients, and may thus be negatively affected by the exclusion-proneness of BPD patients. The aim of this pilot study was to examine the predictive value of threat of social exclusion for the therapeutic alliance in MBT-G. In the first part of the study, social exclusion was experimentally induced in 23 BPD inpatients and 28 healthy subjects using the Cyberball paradigm, a virtual ball tossing game. The evoked level of threat was measured with the Need-Threat Scale (NTS) which captures four dimensions of fundamental human needs, i.e., the need for belongingness, for self-esteem, for control, and for a meaningful existence. In the second part of the study, therapeutic alliance was measured on three dimensions, the therapists, the fellow patients and the group as a whole, using the Group-Questionnaire (GQ-D). BPD patients scored higher in their level of threat according to the NTS in both, the inclusion and the exclusion condition. The level of threat after exclusion predicted impairments of the therapeutic alliance in MBT-G. It was associated with more negative relationships, lower positive bonding and a lower positive working alliance with the fellow patients and lower positive bonding to the group as a whole whilst no negative prediction of the alliance to the therapists was found. Consequently, our translational study design has shown that Cyberball is an appropriate tool to use as an approach for clinical questions. We further conclude that exclusion-proneness in BPD is a critical feature with respect to alliance in group treatments. In order to neutralize BPD patients’ exclusion bias, therapists may be advised to provide an “inclusive stance,” especially in initial sessions. It is also recommendable to strengthen patient to patient relations.

## Introduction

Interpersonal problems are a crucial feature of borderline personality disorder and underlie other straining characteristics of the disease, such as self-harming behavior, affective dysregulation, and impaired social functioning in the long-term course (BPD; Stiglmayr et al., 2005; Gunderson, 2007; Zanarini et al., 2007; Skodol, 2008; Gunderson, 2011; Livesley, 2012; Dammann et al., 2016). Evidence further suggests a close association of interpersonal problems with a particular sensitivity to rejection (Staebler et al., 2011a; Herpertz and Bertsch, 2014) which might distinguish BPD from other personality disorders (Butler et al., 2002). Consequently, rejection sensitivity has been proposed as a target of therapeutic interventions (Renneberg et al., 2012; Bungert et al., 2015).

The Cyberball task is a well-established experimental paradigm to simulate rejection through social exclusion, i.e., ostracism (for a meta-analysis see Hartgerink et al., 2015). It is a computer-based ball tossing game, in which participants are excluded by virtual teammates after having been included before (Williams et al., 2000). By definition, the exclusion condition results in a threat of fundamental human needs (need threat) which are the need to belong, the need for self-esteem, the need for perceived control and the need for a sense of meaningful existence (Baumeister and Leary, 1995; Williams, 2007; Smart Richman and Leary, 2009).

Numerous experimental studies have shown aversive reactions of BPD patients after rejection. For this purpose, most of the previous studies simulated ostracism through the exclusion condition of the Cyberball paradigm with significant emotional and behavioral consequences, including neurobiological investigations (Ruocco et al., 2010; Lawrence et al., 2011; Staebler et al., 2011b; Renneberg et al., 2012; Dixon-Gordon et al., 2013; Chapman et al., 2014, 2015; Gutz et al., 2015, 2016; Ernst et al., 2017; Savage and Lenzenweger, 2017). Some of these studies revealed that feelings of exclusion in BPD were evoked similarly in the exclusion and in the inclusion condition (Staebler et al., 2011a; Renneberg et al., 2012; Domsalla et al., 2014). Further, De Panfilis et al. (2015) have shown that only during “over-inclusion”, i.e., providing more ball tosses to participants than to co-players, BPD patients did not display a negative emotional reaction. In sum, evidence suggests a characteristic exclusion-prone interpretation bias (“exclusion-bias”) in BPD with stronger emotional and behavioral reactions than comparison groups (Lobbestael and McNally, 2016).

Yet, it remains unclear whether the effects of virtual exclusion may be transferred to real interpersonal experiences of BPD patients with peers or attachment figures, such as significant others, in treatment conditions (Ayduk et al., 2008; Lis and Bohus, 2013).

Evidence-based treatments for BPD, e.g., dialectical-behavioral therapy (DBT), schema-focused therapy (SFT), and mentalization-based therapy (MBT) utilize group components. Its beneficial effects on social perception and interpersonal skills in various settings, e.g., outpatient and inpatient treatment, have been outlined (Farrell et al., 2009; Bateman and Fonagy, 2010; Bloom et al., 2012; Karterud and Bateman, 2012; Laurenssen et al., 2014; Kvarstein et al., 2015; Linehan and Wilks, 2015; Dimaggio and Brune, 2016; Fassbinder et al., 2016; Kramer et al., 2016; Antonsen et al., 2017; Edel et al., 2017).

However, an atmosphere of safety including trustful relationships and reliable social bonds is not easily granted in group psychotherapy for BPD, even though it is particularly critical for these patients (Fonagy and Allison, 2014; Sagen Inderhaug and Karterud, 2015; Lonargain et al., 2016). Although therapeutic alliance is regarded a robust predictor for therapy outcome in BPD, empirical knowledge about therapeutic alliance in disorder-specific group psychotherapy is limited (Barnicot et al., 2012; Richardson-Vejlgaard et al., 2013; Bedics et al., 2015; McMain et al., 2015). Alliance in groups is multidimensional and comprises multiple relational factors, such as the relation to the group as a whole, patient-to-therapist and patient-to-patient relationships (Burlingame et al., 2011).

It appears highly plausible that the “exclusion bias” compromises alliance in group psychotherapy, especially during initial sessions, but – to our best knowledge – no study has addressed this question yet. However, if true, particular therapeutic strategies might be required. Consequently, the aim of our pilot study was to explore predictive value of BPD patients’ exclusion-proneness toward the quality of alliance in group psychotherapy. For this purpose, Cyberball-induced threat to human needs was determined in BPD patients and healthy participants (HP). Patients’ level of threat under exclusion was then linked to patient-rated measures of therapeutic alliance – in terms of relations to therapists, fellow patients, and the group as a whole – after the 6th session of mentalization-based group therapy (MBT-G).

Based on previous studies, we assumed a higher need threat after exclusion than after inclusion for all subjects. This effect was expected to be stronger in BPD patients. We further hypothesized a negative association between the need threat score after exclusion and all dimensions of alliance in MBT-G.

## Materials and Methods

### Participants

The sample included 23 BPD patients and 28 HP. All subjects participated in the study between December 2014 and January 2016. The study is part of a larger investigation on clinical and neurobiological predictors of treatment in BPD (BION Basel). It was carried out in accordance with the recommendations of the Ethics committee of North West Switzerland (EKNZ) with written informed consent from all subjects in accordance with the Declaration of Helsinki. The study was approved by the EKNZ (EKNZ: PB_2017-00645 - 2014/078).

BPD patients were recruited during the standard admission procedure for a multimodal personality disorder specific 12-week residential inpatient treatment program in a specialized unit of the Psychiatric University Hospital (UPK) Basel. BPD Patients were admitted on the basis of severe clinical impairment, including chronic self-harming behavior, suicide attempts, comorbidities or sustained social withdrawal. Inclusion criteria were diagnosis of BPD according to the Structured Clinical Interview for DSM-IV Axis-II Disorders (SCID-II, First et al., 1997), age over 18 years and informed consent. Exclusion criteria were substance abuse 1 week before admission, psychotic symptoms and mental retardation. The SCID-II shows excellent inter-rater reliability (Lobbestael et al., 2011) and that was conducted by experienced clinical psychologists and psychiatrists within the first 2 weeks after admission. All 23 BPD diagnoses were confirmed in case discussions with senior psychiatrists to ensure their validity. Comorbidities were assessed using the SCID-II and the Structured Clinical Interview for DSM-IV Axis-I Disorders (SCID-I, First et al., 1996). For sociodemographic data of BPD patients see **Table 1**; for clinical data of BPD patients see **Table 2**.

**Table 1:** Sociodemographic data for BPD patients and healthy participants (HP).

	BPD (*n* = 23)	HP (*n* = 28)	Statistic
Age in years *M* (*SD*)	27.9 (9.5)	25.7 (6.2)	*t*(36.29) = 0.96 *p* = 0.344
Education in years *M* (*SD*)	12.8 (1.7)	15.8 (2.9)	*t*(44.44) = -4.59 *p* < 0.001^∗∗^
**Gender *n* (%)**			
Female	20 (87.0)	24 (85.7)	Fisher‘s Exact *p* = 0.613
Male	3 (13.0)	4 (14.3)	
**Job situation *n* (%)**			
Employed, students	12 (52.2)	28 (100.0)	Fisher‘s Exact *p* < 0.001^∗∗^
Unemployed	11 (47.8)	0 (0)	
**Marital Status *n* (%)**			
Marriage, Partnership	11 (47.8)	11 (39.3)	*Chi^2^*(1) = 0.85 *p* = 0.357
Single	12 (52.2)	17 (60.7)	
**Living situation *n* (%)**			
Living alone	6 (26.1)	5 (17.9)	*Chi^2^*(1) = 0.51 *p* = 0.477
Living with others	17 (73.9)	23 (82.1)	


*Using independent sample *t*-tests, Fischer‘s Exact tests and Chi^*2*^-test, BPD = Borderline Personality Disorder, HP = Healthy Participants, ^∗^*p* < 0.05, ^∗∗^*p* < 0.01.*

**Table 2:** Clinical data for BPD patients (*n* = 23).

	*n* (%)
**Axis I disorder (SCID-I)**	
Major Depression	8 (34.8)
Anxiety Disorders	2 (8.7)
Substance-related Disorders	6 (26.1)
Eating Disorders	3 (13.0)
Somatoform Disorders	2 (8.7)
**Axis II disorder (SCID-II)**	
None	12 (52.2)
One	7 (30.4)
Two	3 (13.0)
Three	1 (4.3)
**Regular medication**	16 (69.6)


*BPD = Borderline Personality Disorder, SCID = Structured Clinical Interview for DSM Disorders (SCID I and II; Wittchen et al., 1997). Regular medication refers to psychopharmacological treatment on a daily basis according to the APA treatment guidelines for BPD and was supervised by psychiatric consultations.*

The inpatient treatment program for the BPD patients consisted of a modified Mentalization-based group psychotherapy (MBT-G; Karterud and Bateman, 2012; Karterud, 2015) based on the quality manual for MBT (Bateman et al., 2012). Patients joined the ongoing group therapy with three sessions of 75 min per week in their 2nd week after admission to the unit. According to institutional requirements the group consisted of 12–14 participants. Due to the slow open access, on average one new patient per week joined the group after another patient had left. Since the group comprised patients from the same unit, only patients knew each other before their first session of MBT-G. Patients did not receive a MBT introductory group (MBT-I, Karterud and Bateman, 2012; Karterud, 2015) beforehand but were introduced to MBT-G during the pre-admission procedure and in an individual session with the principal group therapist during the first week after admission. Additionally, they were officially welcomed in their first group session according to MBT-G principles. The group was conducted by the senior psychiatrist of the unit and an advanced clinical psychologist or resident acting as a co-therapist. Therapists were MBT trained and regularly supervised by a recognized MBT practitioner on the basis of videotaped MBT-G sessions using the MBT adherence scale (Karterud et al., 2013). The treatment consisted of further main components, such as two weekly sessions of individual psychotherapy for 30 min, provided by experienced clinical psychologists or residents who did not function as group therapists. Further, patients received 2 weekly sessions of clinical management by primary nurses and psychiatric consulting including optional prescription of medication. Moreover, expressive therapies, such as art therapy, music therapy and body work therapy were integrated into the program. To assure the integration of all treatments, the MBT-informed case formulations were discussed and updated during weekly meetings with all involved therapists, led by the senior psychiatrist (Bateman and Fonagy, 2016; Euler and Walter, 2018).

The HP group consisted of non-clinical subjects who were recruited by an advertisement on the website of the University of Basel. All HPs have never suffered from psychiatric or psychological disorders according to a semi-standardized psychiatric interview and the SCID Patient Screen Questionnaire (First et al., 2001). For sociodemographic data of HPs see **Table 1**.

### Study Design

The study consisted of two parts: In the first part, both BPD patients and HP played the Cyberball task. The HP group was used as a control group to illustrate the divergent BPD response to the exclusion condition in order to confirm the clinical applicability of the task.

**FIGURE 1:**
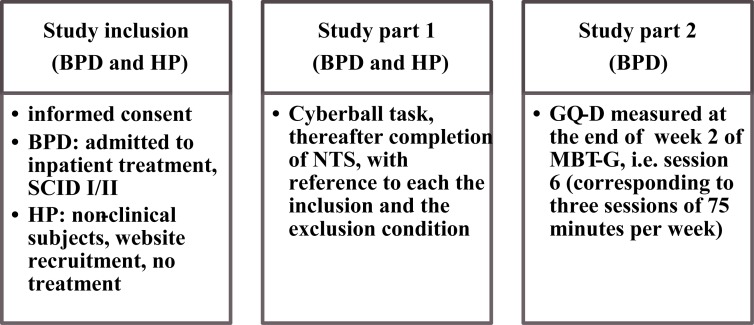
Study design. BPD = Borderline Personality Disorder, HP = healthy participants; SCID = Structured Clinical Interview for DSM Disorders (SCID I and II; Wittchen et al., 1997); NTS = Need Threat Scale (Jamieson et al., 2010); GQ-D = Group Questionnaire, German version (Bormann et al., 2011), MBT-G = Mentalization-based group psychotherapy (Karterud, 2015).

The second part of the study was administered to BPD patients only. Their responsiveness (NTS) to the exclusion condition in the first part of the study was associated to therapeutic alliance as measured by the Group Questionnaire (GQ) after the 6th session of MBT-G. Patients put the anonymized GQ in an envelope and handed it over to the staff. **Figure 1** illustrates the study procedure.

### Cyberball Task

The Cyberball task is a virtual ball tossing game developed by Williams and Jarvis (2006). The subject was told that he would be participating in a mentalization task with two other real-life players and that they were connected to each other via the Internet. In reality, these players were virtual and experimentally manipulated. The respective subject saw one player on the left and a second player on the right side of the computer screen. A picture of the subject had been taken before the experiment and was set as the third player in the game. Three rounds were conducted. In the first round the subject did not receive the ball and only watched the ball being tossed between the two other players. He was told that since the internet connection had broken down he was unable to receive the ball. In the second round (inclusion condition), after being told that the internet connection was reestablished, the subject received the ball 30 times, thus as often as the other players. In the third round (exclusion condition), the subject received the ball only five times. In each condition, the order of the ball tosses was randomized.

Directly after the experiment, all subjects (BPD patients and HP) filled out the NTS (Jamieson et al., 2010) twice, once for the inclusion condition and once for the exclusion condition. For manipulation check, after the experiment, subjects estimated on a ten point Likert scale from 0 to 100%, how often they received the ball in each round and they were asked on a five point Likert scale from 1 (very unlikely) to 5 (very likely) how much they believed to play with a real player. During the debriefing procedure, the story that the other players were computerized was uncovered.

### Measures

#### Need Threat Scale

The Need Threat Scale (NTS; Jamieson et al., 2010) assesses the extent of threat to human needs on four subscales (“Need for belongingness,” “Need for self-esteem,” “Need for control,” and “Need for meaningful existence”). Each subscale consists of five items rated on a seven point Likert scale from 1 (do not agree) to 7 (agree). An overall mean index (NTS-OI) can be computed (Riva et al., 2011). A lower NTS-OI score indicates a higher need threat. The internal consistency in our sample was given (NTS-OI inclusion BPD group Cronbach‘s α = 0.70, HP group Cronbach‘s α = 0.51; NTS-OI exclusion BPD group Cronbach‘s α = 0.74, HP group Cronbach‘s α = 0.79). In current literature the NTS shows a satisfactory internal consistency (Cronbach‘s α = 0.78; Jamieson et al., 2010).

#### Group Questionnaire

The GQ is a self-report questionnaire and was developed to quantify three dimensions of relations in group therapy from the participants’ point of view (Bormann et al., 2011). These dimensions are the relationship between patient and group therapist(s), the relationship between patient and fellow patients as well as the relation to the group as a whole. The first two dimensions are each operationalized by the three scales “positive bonding” (e.g., “I felt that I could trust the group leaders during today’s session”, “I felt that I could trust the other group members during today’s session”) “positive working” (“The group leaders and I agree about the things I will need to do in therapy”, “The other group members and I agree about the things I will need to do in therapy”) and “negative relationship” (“The group leaders did not always understand the way I felt inside”, “The other group members did not always understand the way I felt inside”). The last dimension is operationalized by the scales “positive bonding” (“The group members accept one another”) and “negative relationship” (“There was tension and anxiety between the members”). The questionnaire includes 30 items with seven point Likert scales from 1 (do not at all agree) to 7 (agree very much). On each dimension, for each given subscale, a mean score was calculated. All three scales show a satisfactory internal consistency (negative relationship Cronbach‘s α = 0.74, positive working Cronbach‘s α = 0.91, positive bonding Cronbach‘s α = 0.77). In current literature the internal consistency of the three scales is located between Cronbach‘s α = 0.70 and Cronbach‘s α = 0.92 (Bormann et al., 2011).

### Statistical Analysis

For the comparison of sociodemographic variables between BPD and HP group, we used independent sample *t*-tests, Fischer‘s Exact tests and Chi^2^-test.

For the first part of the study, we have set NTS-OI as the dependent variable and conducted a mixed analysis of variance (ANOVA): group (BPD vs. HP) x condition (inclusion vs. exclusion) for the comparison of inclusion and exclusion condition between the two groups. The Shapiro-Wilk test showed for all used scores and for both groups a normal distribution (*p* > 0.05). Homogeneity of the error variances (Levene‘s test, *p* > 0.05), of the covariance’s (Box‘s test, *p* > 0.05) as well as sphericity were given.

For the second part of the study, the association between NTS-OI after the exclusion condition of Cyberball task as predictor and the GQ subscales as outcome variables was investigated by performing multiple linear regression analyses. We controlled for the NTS-OI after the inclusion condition and for patient’s medication in these models. In each model, the independence according to the Durbin-Watson statistic, homoscedasticity and normally distribution of the residuals were given.

We set a significance threshold of *p* < 0.05 for all analyses. The Statistical Package for Social Sciences (SPSS 24) was used for all statistical analyses.

## Results

### Descriptive Analysis

**Table 1** shows the sociodemographic data of the BPD and HP groups. Results revealed a significant group difference in education [*t*(44.44) = -4.59, *p* < 0.001] and job situation (Fisher‘s Exact *p* < 0.001).

**Table 2** shows the clinical data of the BPD patients. According to the SCID-I, eight patients had a comorbid major depression, six had a comorbid substance-related disorder and five patients suffered from another Axis I disorder (e.g., eating disorder). According to the SCID-II, seven patients had one, three patients had two and one patient had three comorbid personality disorders. Before and during the study, 16 patients had been taking regular medication.

### Manipulation Check of the Cyberball Task

In the baseline condition, both groups reported 0% (*SD* = 0) ball tosses. In the inclusion condition, BPD patients estimated 46.2% (*SD* = 22.8) and the HP group estimated 39.2% (*SD* = 12.6) received ball tosses. In the exclusion condition, BPD patients assumed 12.8% (*SD* = 9.4) and the HP group 10.7% (*SD* = 7.2).

A group comparison with a mixed analysis of variance (ANOVA, group (BPD vs. HP) × condition (inclusion vs. exclusion)) revealed that the ball-receiving-estimations of the condition did not depend on the group [*F*(1.49) = 1.25, *p* = 0.269, partial η^2^ = 0.03]. There was no significant difference between the groups [*F*(1,49) = 1.96, *p* = 0.168, partial η^2^ = 0.04]. Both groups showed a significantly higher ball-receiving-estimation rate in the inclusion condition than in the exclusion condition [*F*(1.49) = 200.51, *p* < 0.001, partial η^2^ = 0.80].

The result matches with the distribution of the experimentally manipulated ball tosses in each condition. On average, BPD patients estimated 3.0 (*SD* = 1.3) and the HP group 3.2 (*SD* = 1.1) in believing the cover story. There was no significant difference between the groups [*t*(45) = -0.53, *p* = 0.60].

### Group Comparison

**Table 3** shows the descriptive mean scores of NTS-OI for each condition and for each group.

**Table 3:** Mean and standard deviations of Cyberball NTS-OI inclusion and NTS-OI exclusion for BPD patients and healthy participants (HP).

	NTS-OI Inclusion		NTS-OI Exclusion	
		
	*M*	*SD*	*M*	*SD*
BPD (*n* = 23)	3.69	0.58	2.51	0.65
HP (*n* = 28)	4.06	0.38	2.76	0.58


*NTS-OI = Need Threat Scale - overall mean index, BPD = Borderline Personality Disorder, HP = Healthy Participants, a lower NTS-OI value indicates a higher need threat.*

**FIGURE 2:**
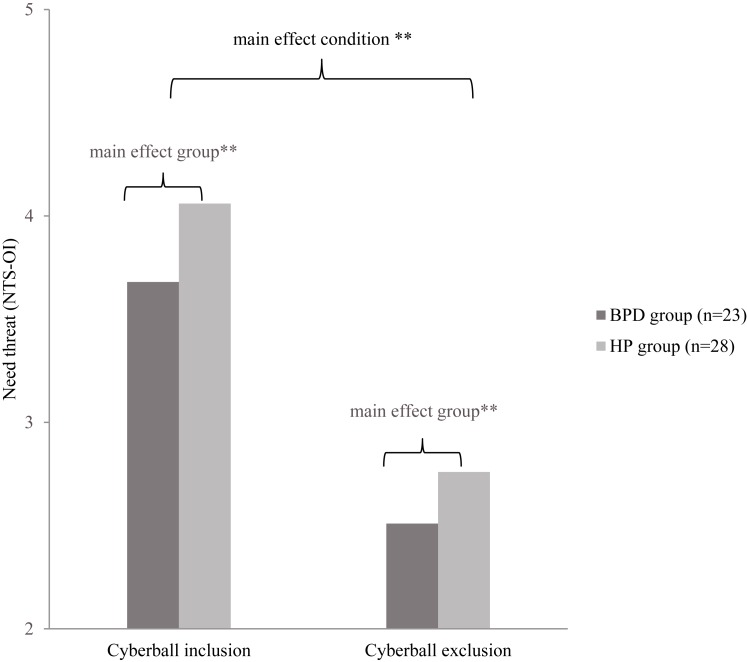
NTS-OI = Need threat scale - overall mean index, BPD = Borderline Personality Disorders, HP = Healthy Participants. NTS-OI scores in Cyberball inclusion and exclusion condition for BPD and HP. A lower NTS-OI value indicates a higher need threat. ^∗∗^*p* < 0.01.

The mixed ANOVA revealed a significant main effect of condition. This means that there was a significant difference between the NTS-OI inclusion and NTS-OI exclusion condition independent of the group, i.e., both groups showed higher need threat (NTS-OI) in the exclusion than in the inclusion condition [*F*(1,49) = 143.39, *p* < 0.001, partial η^2^ = 0.75). Further, there was a significant main effect of group, independent of condition. The BPD group showed a higher need threat level than the HP group in both conditions [*F*(1,49) = 7.52, *p* = 0.009, partial η^2^ = 0.13]. There was no significant interaction between condition (exclusion vs. inclusion) and group (BPD vs. HP) which indicates that the condition effect and the group effect did not depend on each other [*F*(1,49) = 0.36, *p* = 0.554, partial η^2^ = 0.01]. **Figure 2** illustrates the described effects.

After adding the factors which showed a significant group difference (education years and job situation) to the mixed ANOVA model, we found no significant interaction effects [condition × job situation: *F*(1,49) = 1.32, *p* = 0.257; condition x education years: *F*(1,49) = 0.85, *p* = 0.362; condition × group: *F*(1,49) = 0.10, *p* = 0.536].

### NTS and GQ in BPD

The multiple regression analysis revealed that NTS-OI in the exclusion condition significantly predicted alliance to fellow patients and the group as a whole. The effects remained significant when controlling for the NTS-OI after the inclusion condition and for patient’s medication. BPD patients with higher need threat after exclusion showed higher negative relationships, lower positive bonding and lower positive working alliance to fellow patients as well as a lower positive bonding to the group as whole. For the significant associations, NTS-OI explained between 10 and 30% of the variance, which indicates a moderate goodness-of-fit of the models (Ellis, 2010). There was no significant association between NTS-OI and the alliance to the group therapist. **Table 4** shows all results of the regression analysis.

**Table 4:** Multiple Regression Analysis of Group Questionnaire (GQ) and NTS-OI after exclusion Cyberball condition controlling for inclusion condition and medication in BPD group (*n* = 23).

	β	adjusted *R^2^*	*p*
**Alliance to fellow patients Negative relationships**			
Model 1		0.14	
NTS-OI after exclusion	-0.88		0.043*
Model 2		0.10	
NTS-OI after exclusion	-0.88		0.050
NTS-OI after inclusion	-0.04		0.935
Model 3		0.10	
NTS-OI after exclusion	-1.00		0.034*
NTS-OI after inclusion	-0.16		0.748
Medication	-0.64		0.316
**Alliance to fellow patients Positive bonding**			
Model 1		0.21	
NTS-OI after exclusion	0.69		0.015*
Model 2		0.19	
NTS-OI after exclusion	0.68		0.020*
NTS-OI after inclusion	0.17		0.576
Model 3		0.25	
NTS-OI after exclusion	0.79		0.008**
NTS-OI after inclusion	0.29		0.350
Medication	0.61		0.123
**Alliance to fellow patients Positive working**			
Model 1		0.16	
NTS-OI after exclusion	0.68		0.033*
Model 2		0.12	
NTS-OI after exclusion	0.68		0.035*
NTS-OI after inclusion	-0.10		0.767
Model 3		0.12	
NTS-OI after exclusion	0.77		0.025*
NTS-OI after inclusion	-0.02		0.955
Medication	0.44		0.336
**Alliance to group as whole Negative relationships**			
Model 1		-0.03	
NTS-OI after exclusion	-0.25		0.501
Model 2		-0.05	
NTS-OI after exclusion	-0.28		0.469
NTS-OI after inclusion	0.31		0.471
Model 3		-0.06	
NTS-OI after exclusion	-0.37		0.361
NTS-OI after inclusion	0.22		0.621
Medication	-0.49		0.396
**Alliance to group as whole Positive bonding**			
Model 1		0.16	
NTS-OI after exclusion	0.58		0.035*
Model 2		0.11	
NTS-OI after exclusion	0.58		0.040*
NTS-OI after inclusion	-0.01		0.978
Model 3		0.30	
NTS-OI after exclusion	0.74		0.007**
NTS-OI after inclusion	0.16		0.580
Medication	0.88		0.022*
**Alliance to group therapist Negative relationships**			
Model 1		-0.03	
NTS-OI after exclusion	-0.22		0.591
Model 2		-0.06	
NTS-OI after exclusion	-0.24		0.562
NTS-OI after inclusion	0.29		0.540
Model 3		-0.06	
NTS-OI after exclusion	-0.13		0.766
NTS-OI after inclusion	0.40		0.406
Medication	0.62		0.317
**Alliance to group therapist Positive bonding**			
Model 1		-0.04	
NTS-OI after exclusion	0.17		0.667
Model 2		-0.09	
NTS-OI after exclusion	0.16		0.684
NTS-OI after inclusion	0.06		0.896
Model 3		-0.10	
NTS-OI after exclusion	0.07		0.870
NTS-OI after inclusion	-0.04		0.936
Medication	-0.52		0.391
**Alliance to group therapist Positive working**			
Model 1		0.03	
NTS-OI after exclusion	0.55		0.225
Model 2		-0.01	
NTS-OI after exclusion	0.56		0.222
NTS-OI after inclusion	-0.22		0.661
Model 3		-0.01	
NTS-OI after exclusion	0.69		0.149
NTS-OI after inclusion	-0.09		0.861
Medication	0.71		0.296


*NTS-OI = Need Threat Scale - overall mean index, GQ = Group Questionnaire, BPD = Borderline Personality Disorder, ^∗^*p* < 0.05, ^∗∗^*p* < 0.01.*

## Discussion

Our study investigated the effect of ostracism, in terms of experimentally induced social exclusion, on threat to fundamental human needs in BPD patients and healthy subjects. We further examined the predictive value of the exclusion-related need threat with various dimensions of therapeutic alliance in MBT-G.

All participants showed higher need threat after the social exclusion condition compared to the inclusion condition. BPD patients showed higher need threat than the HP group after both conditions. Further, BPD patients who experienced higher need threat after exclusion showed higher negative relationships, lower positive bonding, and lower positive working alliance to fellow patients as well as a lower positive bonding to the group as a whole in primary sessions of MBT-G. In other words, BPD new arrivals with a higher exclusion-proneness had more difficulty establishing social connectivity, i.e., supportive and cooperative relations, with other group members. Interestingly, these effects solely referred to fellow patients and to the group as a whole but not to the therapists.

Our findings are in line with previously published studies and thus support evidence that BPD patients are increasingly prone to feeling threatened by exclusion in social participation. (Staebler et al., 2011a; Renneberg et al., 2012; Domsalla et al., 2014). On the basis of our study, we further argue that the psychological reactivity to experimental, i.e., virtual, ostracism qualified to forecast the nature of a real interpersonal interaction. Thus, the Cyberball task has turned out to be an appropriate tool to approach a clinical question.

Given the evidence for an exclusion-prone interpretation bias of BPD patients, it is tempting to speculate that the ability to establish positive relations in group therapy is more restrained the more exclusion-prone patients perceive themselves as potentially threatened. Their interpretation bias in social encounters has been conceptualized as a failure of mentalizing with an (over-)attribution of malevolent intentions to others and a lack of discrimination between self and other (Sharp et al., 2011; Domsalla et al., 2014; Herpertz and Bertsch, 2014). As such, the breakdown of mentalization might induce a vicious circle: Once patients feel ostracized, they may not recognize any agreeable interactional cues by others anymore and perceive neutral mimicry as aversive (Staebler et al., 2011a; Savage and Lenzenweger, 2017). Feelings of exclusion are therefore perceived in “psychic equivalence” (i.e., an inner reality is perceived as “over”-real, Bateman and Fonagy, 2016).

However, taking the discrepancy between the relational valuation of fellow patients and therapists into account, even the exclusion-prone patients appeared to be capable of seeing the therapists not as malevolent. This might be unsurprising, since MBT-G therapists are supposed to engage extensively in the therapeutic alliance, which is similar to other group therapies for BPD, such as SFT group therapy and DBT skills-training. An active, responsive and reliable therapeutic stance has been described as a hallmark of all empirically supported therapies for BPD (Barnicot et al., 2012; Bateman et al., 2015; McMain, 2015; Kramer, 2017). For instance, to deal with patients’ mistrust in social encounters, therapists in MBT deliver ostensive (non-)verbal cues to make patients feel sufficiently valued and addressed (Fonagy et al., 2015). This fairly “inclusive” – to some extent perhaps even “over-inclusive” – attitude may partially explain why patients did not feel uncomfortable toward the therapists in our study. Yet, we have to raise the question whether therapists took patient to patient relations into sufficient consideration. MBT-G includes specific interventions for the linking of patients, such as “connecting” and “siding” (i.e., explicitly backing patients in weak positions, Karterud and Bateman, 2012; Bateman and Fonagy, 2016; Euler and Walter, 2018). Further, MBT genuinely focusses on the enhancement of mentalizing which should support patients to revise negatively biased perceptions of others (Luyten and Fonagy, 2015; Dimaggio and Brune, 2016). However, the therapists in our study did not succeed in establishing a satisfying connection of exclusion-prone new arrivals with their fellow patients. It remains an open question whether the MBT-G principles were not appropriate in dealing with this issue, which must otherwise be seen as a fairly high demanding endeavor. On the other hand, the therapists might have performed these interventions insufficiently during the study. Further, since we solely assessed alliance in the primary sessions we do not know whether these patients developed a more favorable relatedness to their peers in the further course.

Meanwhile, our results may be interpreted by consulting other approaches. For instance, from a psychodynamic point of view, the relational discrepancy may be evaluated as a split off of object-relations with an (initial) idealization of the therapists whilst aversive representations would be projected into the peers. As a consequence, other, non-MBT interventions, e.g., transference interpretation, which is the hallmark of transference-focused psychotherapy (TFP), would have possibly been more effective (Hoglend et al., 2011; Clarkin and Kernberg, 2015).

Overall, establishing a trustworthy, cooperative and cohesive relational climate in group therapy with BPD patients remains a highly challenging endeavor – especially in primary sessions and when patients feel easily threatened by exclusion. A precise adjustment to BPD patients’ current mental state, including their distorted perception of others as excluding, is difficult to achieve – even with interventions that specifically target this issue, e.g., in MBT-G. This problem is underlined by BPD patients’ constant fluidity of mind states with a perpetual instability of individual needs (Levy et al., 2010; Kramer, 2017). Further, some patients might respond well to highly structured groups, e.g., DBT skills-training, but struggle in groups with a stronger interpersonal, i.e., attachment focus, e.g., MBT-G. Thus, patients who are dedicated to group treatments have to be assessed carefully and differential indications have to be taken into consideration. Conceivably, patients with disorganized attachment or highly distorted object relations – displaying a high risk for a critical exclusion bias – might initially require individual treatment even if group therapy is suggested. Such individualized treatment suggestions are in line with current recommendations for the further development of evidence-based treatments for BPD. Whilst they have shown comparably favorable effects in general, we do not know much about individual needs and therapy response of BPD patients with specific psychological problems, such as exclusion-proneness (Cristea et al., 2017; Kramer, 2017; Links et al., 2017).

### Limitations and Recommendations for Further Research

The strength of our study is the translational design. Translational studies with behavioral experiments are considered as appropriate to provide substantial information for a development of personalized treatment approaches in BPD (Sharp and Kalpakci, 2015; Kramer, 2017). In our study, the virtual “exclusion bias” is not only transferred into a real interpersonal exchange but even used to investigate a critical clinical question. The study further enriches research within the under-investigated field of group psychotherapy.

Our study has several limitations. First and foremost, our results have to be considered as explorative because of the small sample size. With respect to the Cyberball experiment, the simultaneous recall of need threat for both conditions might have confounded their discrimination. However, our results do not diverge from several previously published studies.

We neither provided a clinical control group nor a control condition. Including a clinical control group would have been interesting to provide the opportunity to proceed with both groups into the second part of the study. Moreover, patient or setting variables have potentially confounded the results. The subjects were inpatients and thus may represent a severely impaired subpopulation of BPD that is exceptionally difficult to treat. However, their rate of comorbidities and level of social functioning (e.g., employment, partnership) appears moderate. Further, the group had 12–14 participants which are more than proposed in the genuine model (6–9) and patients did not receive the original structured introduction to the treatment (MBT-I, Bateman and Fonagy, 2016). These variations may also have confined the capacity to “include” new arrivals adequately. Further, it seems worth mentioning that the face-to-face encounter with the group therapist in the introductory appointment probably promotes trust resulting in a benefit for the relationship to him in the first group meeting. Since patients were residents in the same unit, negatively biased relational patterns within the patients may partly be attributed to interpersonal exchange outside the group sessions, for instance before admission to the group. MBT-G was carefully supervised via videotapes to confirm adherence to the model but we did not use a formal adherence protocol with randomized controls by independent raters (Karterud et al., 2013; Karterud, 2015). Model-specific interpretations must therefore be handled cautiously. Since we did not control for symptom severity, the specificity of the link between high need threat and impairment of the therapeutic alliance is not certain. According to our explorative statistical analysis we cannot rule out the problem of the alpha-error inflation by performing multiple tests.

Taking these limitations into account, we regard our results and conclusions as highly preliminary. On the basis of our pilot study, we permit to outline some recommendations for further research. In general, more translational research on a basis of behavioral experiments with larger samples is needed to foster the explanatory potential of basic research for clinical questions. Other experimental predictors (e.g., the reading the mind in the eyes test, Baron-Cohen et al., 2001) of therapeutic alliance in group therapy might be of further interest. A comparison of different treatment models and their success in coping with patients’ exclusion bias would possibly outline their strengths and shortcomings. To ensure therapists conformity, structured adherence assessments are highly recommended. Direct measures of exclusion-related features in group therapy and trait characteristics, such as rejection sensitivity, as predictors or moderators might further outline whether patients with high scores in these measurements actually have the potential for favorable outcomes in group therapy. If not, such measures may alternatively serve as counter-indicators in clinical assessments.

## Conclusion

Linking the Cyberball task with a naturalistic treatment condition to unfold the implication of BPD patients’ exclusion bias in group therapy has proven to be a promising endeavor. Our study highlights the significance of the patient to patient alliance in group therapies for BPD patients. It is advised to keep their exclusion-proneness in mind during clinical practice, training, and supervision of group therapies. Giving patients a careful introduction before admission and implementing small groups may be helpful for their successful reception. Further, the contemplation of differential indications for different treatment approaches, such as setting- (group vs. individual) or model-related (e.g., DBT skills-training vs. MBT-G) factors, might be crucial for patients with a high risk for an exclusion-biased distortion in the therapeutic alliance. More research is needed to verify which interventions successfully focus on patient to patient relations in order to establish a holding environment for exclusion-prone subjects. In providing such, group therapy may deliver excellent conditions to override the devastating constraints of social participation in BPD.

## Author Contributions

SE conceived and designed the study, coordinated recruiting of participants and data collection, and wrote all drafts of the manuscript. JW coordinated recruiting of participants and data collection and implemented, and conducted the Cyberball experiment. MB was responsible for data management and statistical analyses. HL was responsible for final text editing and the submission procedure. DS was involved in the original development of the research project and was responsible for its implementation in the clinical unit. JG was senior methodological supervisor. UL and MW provided overall coordination and supervision of the project. All authors listed have made a substantial contribution to the work and approved it for publication.

## Conflict of Interest Statement

The authors declare that the research was conducted in the absence of any commercial or financial relationships that could be construed as a potential conflict of interest.
